# Assessment of a new questionnaire for self-reported sun sensitivity in an occupational skin cancer screening program

**DOI:** 10.1186/1471-5945-8-4

**Published:** 2008-10-24

**Authors:** Jürgen Tacke, Jens Dietrich, Beatrix Steinebrunner, Antonius Reifferscheid

**Affiliations:** 1 Joint Group Practice Dermatology, Bremsstrasse 19, 50969 Cologne, Germany; 2 Medical Corporate Department, Henkel KGaA, Henkelstrasse 67, 40589 Düsseldorf, Germany

## Abstract

**Background:**

Sun sensitivity of the skin is a risk factor for the development of cutaneous melanoma and other skin cancers. Epidemiological studies on causal factors for the development of melanoma must control for sun sensitivity as a confounder. A standardized instrument for measuring sun sensitivity has not been established yet. It is assumed that many studies show a high potential of residual confounding for sun sensitivity. In the present study, a new questionnaire for the assessment of self-reported sun sensitivity is administered and examined.

**Methods:**

Prior to an occupational skin cancer screening program, the 745 participating employees were asked to fill in a questionnaire for self-assessment of sun sensitivity. The questionnaire was developed by experts of the working group "Round Table Sunbeds" (RTS) to limit the health hazards of sunbed use in Germany. A sun sensitivity score (RTS-score) was calculated using 10 indicators. The internal consistency of the questionnaire and the agreement with other methods (convergent validity) were examined.

**Results:**

The RTS-score was calculated for 655 study participants who were 18 to 65 years of age. The correlation of the items among each other was between 0.12 and 0.62. The items and the RTS-score correlated between 0.46 and 0.77. The internal consistency showed a reliability coefficient with 0.82 (Cronbach's alpha). The comparison with the Fitzpatrick classification, the prevailing standard, was possible in 617 cases with a rank correlation of r_s _= 0.65. The categorization of the RTS-score in four risk groups showed correct classification to the four skin types of Fitzpatrick in 75% of the cases. Other methods for the assessment of sun sensitivity displayed varying agreements with the RTS-score.

**Conclusion:**

The RTS questionnaire showed a sufficient internal consistency. There is a good convergent validity between the RTS-score and the Fritzpatrick classification avoiding shortcomings of the prevailing standard. The questionnaire represents a simple, reliable and valid instrument for the assessment of sun sensitivity. The questionnaire can be useful for epidemiological studies as well as for skin cancer prevention. Further development and standardization of sun sensitivity assessments is necessary to strengthen the evidence of epidemiological studies on causal factors of melanoma and other skin cancers.

## Background

Exposure to ultraviolet radiation and certain host factors are associated with skin cancer [1]. Sun sensitivity is one of the risk factors for the development of cutaneous malignant melanoma, basal cell carcinoma and squamous cell carcinoma [2-4]. In epidemiological studies on the identification of causal factors for malignant melanoma, sun sensitivity is expected to have a confounding effect [5]. Obtaining an accurate measurement of sun sensitivity is therefore highly important. However, there exists neither a standardized definition of sun sensitivity nor a standardized measurement. Terms used in connection with sun sensitivity such as "skin type" can also have different meanings [4,6]. The classification by Fitzpatrick has served as a standard for a long time [7]. In a recent review, the Fitzpatrick classification was found to not sufficiently represent sun sensitivity as a risk factor [4]. One of the major problems of the Fitzpatrick classification is the subjective and arbitrary allocation to a class [8-12]. Previous epidemiological studies controlling for sun sensitivity have additionally taken various phenotypic attributes into account. Table 1 shows some studies that measured sun sensitivity by means of different indicators [2,5,13-20]. The indicators were either regarded individually as an independent variable in multivariate analyses or a score was created prior to the multivariate analysis. If only a single indicator for sun sensitivity is taken into account in a multivariate regression model, the problem of misclassification is likely to arise. If several indicators for sun sensitivity with a strong correlation are included in multivariate models as independent variables, the problem of multicollinearity can occur [18].

**Table 1:** Indicators for the measurement of sun sensitivity in recently published studies

Studies	Indicators for sun sensitivity
Swerdlow et al. 1988 [13]	Skin type, hair color, eye color

Westerdahl et al. 2000 [14]	Skin type (repeated exposure), hair color

Veierod et al. 2003 [5]	Skin type (single exposure), skin type (repeated exposure), hair color, eye color

Bataille et al. 2004 [15]	Skin type

Weinstock 1992 [16]	Prediction rule: Skin type, hair color, skin color

Nelemans et al. 1993 [17]	Sun sensitivity summary score: Skin type, skin color, eye color, freckles

Chen et al. 1998 [18]	Cutaneous phenotype index: Skin type, hair color, eye color

Uter et al. 2004 [19]	Constitutional UV sensitivity score: Hair color, freckles

Guinot et al. 2005 [20]	Skin sensitivity to sun exposure score: Hair color, skin color, freckles, tendency for sunburn, tanning ability

Han et al. 2006 [2]	Constitutional susceptibility score: Skin color, hair color, tendency to burn, number of raised moles on arm, (age)

Gallagher found that the existing measurements of sun sensitivity are "crude" and presumed that in many studies risk stratification is performed only insufficiently [21].

Recently, the German Federal Office for Radiation Protection established the working group "Round Table Sunbeds" consisting of experts from science and business to limit the health hazards of sunbed use. The working group developed a questionnaire for the assessment of sun sensitivity which can be found in the attachment to this report [22]. The questionnaire was initially developed for employees of indoor tanning facilities to assess sun sensitivity of their customers in order to adapt the UV-dose to the individual risk.

The purpose of the study is to assess a new questionnaire for self-reported sun sensitivity by determining acceptance, internal consistency and convergent validity in an occupational skin cancer screening program.

## Methods

### Study description

The occupational skin cancer screening program for employees was part of the German skin cancer prevention campaign 2007 [23]. The organization of the skin cancer screening and the recruitment of study participants was done by the Medical Corporate Department (MCD) of the Henkel company in Düsseldorf (Head of MCD: A. Reifferscheid, MD), which is also responsible for several other companies in Düsseldorf. The BKK Essanelle, a health insurance provider and the participating companies sponsored the program. The study is in compliance with the Helsinki Declaration. It was not financially supported. About 9000 employees of different companies work at the plant in Düsseldorf. The employees were informed about the intended skin cancer screening by email and company newsletters. The employees registered for the skin cancer screening at the MCD. Before the skin examination, the participants were asked to voluntarily fill in a questionnaire about their skin sensitivity and their lifelong UV-exposure. The sun sensitivity of the skin was assessed according to the RTS-questionnaire. The fourth question of the questionnaire was slightly modified, because pre-studies had shown that the recommended wording of question 4 remained unclear for many participants (Additional file 1). The RTS-questionnaire is the result of a consensus in the working group "Round Table Sunbeds" (according to personal information by Prof. E.W. Breitbart, Buxtehude, Germany). The authors of this study were neither part of the working group nor involved in the development of the questionnaire.

The study participants were asked about their medical history in a separate part of the questionnaire. 17 study participants made statements that indicated a possible sun allergy, which had never been confirmed by a physician. Therefore, these participants were not excluded from the evaluation.

The RTS-questionnaire consists of ten questions (items) with four possible answers to each question (Likert scale). The possible answers for each item are ordinal scaled. Each possible answer is assigned a value between 1 and 4. The sum of all 10 values from the 10 answers determines the RTS-score. The RTS-score represents the sun sensitivity of the skin. A minimal score of 10 points (high sun sensitivity) and a maximal score of 40 points (low sun sensitivity) can be reached. The RTS-score is compared to the skin type classes by Fitzpatrick and Uter and to the "skin sensitivity to sun exposure score" (SSSE) developed by Guinot [7,19,20]. The skin type classes and the SSSE can be derived from the answers by recoding. For the comparison with the other methods for sun sensitivity measurement, the RTS-score was divided into 4 classes with 7.5 intervals each.

According to Fitzpatrick there are four skin type classes (FP-classes) for the white population. The distribution into four classes is based on answers to two questions: "Do you burn at first average sun exposure?" and "Do you tan at first average sun exposure?" [24]. The answers are categorized according to Fitzpatrick as follows, whereby the immediate pigmentation [question 7] and the pigmentation after repeated UV-exposures are distinguished [question 8]: FP-class 1: sunburn always, tanning never (RTS-questionnaire question 5 answer 1 and question 7 answer 1 = 5/1–7/1); FP-class 2: sunburn always, tanning seldom (5/2–7/1, 5/1–7/2, 5/2–7/2, 5/3–7/2, 5/3–7/1); FP-class 3: sunburn seldom, tanning always (5/2–7/3, 5/3–7/3, 5/4–7/3, 5/2–7/4, 5/3–7/4); FP-class 4: sunburn never, tanning always (5/4–7/4). The classification for pigmentation after repeated sun exposure is done by replacing "7" by "8" in the recoding process mentioned above. In the present study, the corresponding skin type classes could be assigned in 602 cases (immediate pigmentation) or in 617 cases, (pigmentation after repeated UV-exposure). In some cases, the FP-classes did not match, because the participants stated, for example, frequent sunburn, but excellent tanning etc. – these cases could not be assigned to any of the FP-classes.

The skin type classes by Uter are based on two phenotypic indicators: hair color and freckles [19]. The Uter-classes which can be approximately derived from the existing data are: class 1: black hair according to question 9 answer 4 (9/4), class 2: brown hair, no freckles (9/3–2/4), class 3: brown hair, freckles or blond hair without freckles (9/3–2/(1+2+3) or (9/2–2/4)), class 4: blond hair with freckles (9/2–2/(1+2+3)), class 5: red hair (9/1). 655 cases could be assigned to the RTS-score. For the comparison of the categories, the classes 4 and 5 were combined in order to compare them with the 4 categories of the RTS-score.

The SSSE-score by Guinot is based on five questions with several possible answers to each question. The possible answers are assigned coefficients [[20], table 8]. The indicators are hair color, skin color, freckles, tendency for sunburn and ability to tan. The SSSE-score can be derived from the RTS-questionnaire by combining the answer possibilities to the questions 1, 2, 5, 8 and 9 and by "loading" the answer possibilities with the coefficients.

The loadings for the answers to question one (skin color) are: 1/1–1/3 = 0.83, 1/4 = -1.02; question two (freckles): 2/1–2/3 = 1.01, 2/4 = -0.34; question five (tendency to sunburn): 5/1 = 1.45, 5/2 = 0.78, 5/3 = -0.67, 5/4 = -1.23; question eight (ability to tan); 8/1 = 1.09, 8/2 = 0.30, 8/3–8/4 = -1.00; question 9 (hair color): 9/1–9/2 = 1.16, 9/3 = 0.20, 9/4 = -0.87.

The coefficients of the answers to the 5 questions are summed up and after correction with a constant (+4.46) the score-values between 0 and 10 are calculated. A SSSE-score of 10 points represents high sun sensitivity.

### Statistical methods

The statistical analyses were performed with the statistical software environment R version 2.4.1 [25].

The analyses that were performed are based on the assumption that sun sensitivity is a continuous biological attribute. It is assessed by the RTS-questionnaire. The score resulting from the questionnaire represents a quantitative and discrete variable. The RTS-score is based on the sum of ordinal scaled items, meaning that it is scaled ordinally in a narrow sense. However, in this study it is treated like an interval scaled score [26]. The categories of the items are chosen by dermatological experts so that the assumption of a continuum in the ordinal scale is plausible [27]. As an informal rule of thumb, an examination of the ordinal scaled measurement of the Health Related Quality of Life states that the number of categories of the outcome variable should be higher than seven, so that a continuous scale can be applied [27]. The RTS-score can reach 31 discrete values, so that the preconditions for the application of the interval scale are given.

The data are presented in cross tables and box plots. Normality was assessed by displaying the RTS-score data in a normal probability plot. The reliability of the questionnaire was evaluated by calculating the internal consistency (Crohnbach's alpha). Convergent validity was assessed by comparing the RTS-score to other methods (Fitzpatrick classification, Uter classification, SSSE-score).

The strength of associations is presented by the Spearman correlation coefficient. The 95% confidence interval of the mean difference of the RTS-score between females and males was calculated using the t-distribution for independent samples.

## Results

745 patients were examined within two weeks. All study participants were white. The questionnaire was returned in 702 cases. 686 of the returned questionnaires were evaluable. 393 (58%) women and 283 (42%) men aged 18 to 65 participated in the study. In 10 cases, the question about sex was left blank. In table 2, the demographic data of those study participants who made statements about their gender and age are presented. About 40% of the examined study participants were between 36 and 45 years of age. In the age group 26 to 35 years there were relatively more women than men, while in the age group 46 to 55 there were relatively more men than women.

**Table 2:** Frequencies of age groups by gender

		Frequencies (%)	
		
		female N = 392	male N = 282
Age groups	18–25	9	7
in years	26–35	30	19
	36–45	41	38
	46–55	16	30
	56–65	4	6

674 of the 686 participants reported their gender and age.

The distribution of indicators is presented in table 3. Approximately one third of the participants state that their skin sunburns easily and that it shows no or only moderate pigmentation. There are great differences between immediate pigmentation and pigmentation after repeated UV-exposure. Gender differences of more than 10% can be seen for freckles and for sun sensitivity of the face.

**Table 3:** Distribution of categories of the indicators for sun sensitivity by gender*

Indicators	Gender (%)		
	female n = 393	male n = 283	sum n = 676
1. Untanned skin color	n = 385	n = 276	n = 661
reddish	4	4	4
pale	61	51	57
light brown	34	44	38
brown	1	1	1

2. Freckles	n = 384	n = 274	n = 658
many	15	12	13
some	37	27	33
few	26	25	26
none	22	36	28

3. Face: sensitivity to sun exposure	n = 384	n = 275	n = 659
very sensitive	11	6	9
sensitive	28	16	23
normal	49	61	54
insensitive	12	18	14

4. Time until sunburn (in min.)	n = 381	n = 274	n = 655
< 15	12	6	9
15–25	44	41	43
25–40	32	38	34
> 40	13	15	14

5. Frequency of sunburn	n = 382	n = 273	n = 655
always	13	8	11
almost always	25	22	24
often	33	40	35
never	29	30	30

6. Intensity of sunburn	n = 380	n = 275	n = 655
redness, blisters	5	4	5
redness, strong peeling	25	28	26
redness, mild peeling	59	62	60
mild redness	11	6	9

7. Immediate pigmentation	n = 381	n = 275	n = 656
never	7	6	6
hardly ever	27	27	27
often	41	43	42
almost always	24	26	25

8. Pigmentation after repeated exposure	n = 381	n = 273	n = 654
never or hardly ever	1	2	1
slight	36	26	32
progressive	56	65	60
quick	7	8	7

9. Natural hair color	n = 383	n = 273	n = 656
red, red brown	4	5	4
light blond, blond	25	17	22
dark blond, brown	59	57	58
dark brown, black	12	22	16

10. Eye color	n = 383	n = 271	n = 654
light blue, light grey, light green	10	9	9
blue, grey, green	58	61	59
light brown, dark brown	15	11	13
dark brown	18	19	18

*Due to missing data the values do not add up to 676.

Due to missing values, the RTS-score was only calculated in 655 cases. If one of the 10 questions was not answered, the score could not be calculated. The normal probability plot showed a straight line. Based on the visual inspection, it could be assumed that the RTS-score is normally distributed. The distribution had a mean of 26.7 with a standard deviation of 5.1. Females showed slightly increased sun sensitivity with a mean of 26.2 compared to 27.4 for men. Although the 95% confidence interval for the difference of -1.2 (-1.99 to -0.42) did not include zero, the absolute difference is relatively small.

The ten indicators of the RTS-score are not independent from one another. The rank correlations of the items are between 0.12 and 0.62. The tendency for sunburn (question 5) and the ability to tan (question 8) were correlated with r_s _= 0.53. Many associations between the ten indicators are known to exist and could also be described here: Hair color and eye color had a correlation of 0.39, skin color and hair color a correlation of 0.45, skin color and sunburn a correlation of 0.49 and skin color and pigmentation a correlation 0.51. Cronbach's alpha serves as a measure for the internal consistency of the questionnaire. In our study a value of 0.82 was calculated. A value above 0.7 is generally regarded as sufficient, if a wide enough range of items for the construct is assumed.

The categories of the questions were correlated with the RTS-score to evaluate which indicator estimates the RTS-score best (table 4). The skin reaction to repeated sun exposure showed the best representation of the RTS-score applying rank correlation.

**Table 4:** Comparison of indicators to the RTS-score

Indicators for sun sensitivity	Spearman correlation r_s_	Correct classification (%)
1: Untanned skin color	0,66	59
2: Freckles	0,54	43
3: Sensitivity to sun exposure of the face	0,65	59
4: Time until sunburn	0,74	61
5: Frequency of sunburn	0,77	53
6. Intensity of sunburn	0,61	56
7: Pigmentation after single exposure	0,68	56
8: Pigmentation after repeated exposure	0,72	68
9: Natural hair color	0,58	56
10: Eye color	0,46	39
Uter-classification	-0,45	38
Fitzpatrick-classification (single exposure)*	0,62	68
Fitzpatrick-classification (repeated exposure)**	0,65	75
Guinot-Score (SSSE)	-0,88	61

* n = 602, ** n = 617

The first ten indicators are the items of the RTS-questionnaire. The association of the indicators with the RTS-score is demonstrated using the Spearman rank correlation. The last column shows the correct classification of the indicator (four categories) compared to the RTS-score, which is categorized in four groups.

**Figure 1:**
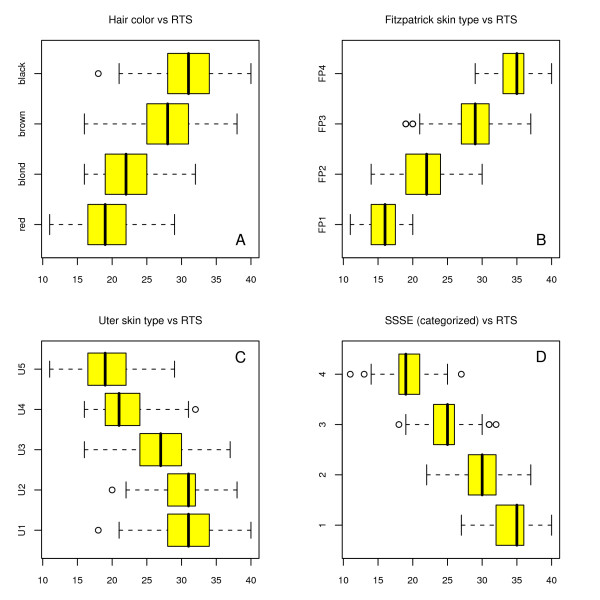
**Relationship between RTS-score and other methods for the measurement of sun sensitivity.** The box plots show the distributions of the RTS-score. **A**: RTS-score by four categories of hair color: 1: red, red brown; 2: light blond, blond; 3: dark blond, brown; 4: black. (n = 655). **B**: RTS-score by skin type classes of Fitzpatrick, pigmentation after repeated exposure (n = 617). **C**: RTS-score by skin type classes of Uter (n = 655). **D**: RTS-score by SSSE-score after categorization of SSSE-score in four groups: 1: SSSE ≤ 2.5; 2: > 2.5 ≥ 5.0; 3: > 5.0 ≤ 7.5; 4: > 7.5 (n = 655).

If the values of the RTS-score are categorized into four groups, the RTS-score categories can be compared with the categories of the answers to the ten questions. The percentage of correct classifications is shown in table 4. If only the 10 indicators of the RTS-questionnaire are taken into account, it is obvious that tanning after repeated sun exposure shows not only a high correlation with a result of 0.72, but also in 68% of cases the answers show the correct classification with regard to the RTS-score. The tendency for sunburn is a better estimate of the RTS-score than hair color. Figure 1A shows the distribution of the RTS-score according to categories of hair color in a box plot. It shows a clear overlapping of the classes: every second value is incorrectly categorized in comparison to the RTS-score.

In 617 cases the following frequencies for the FP-classes (pigmentation after repeated UV-exposure) can be derived from the questionnaire by recoding: FP 1: 1%, FP 2: 31%, FP 3: 62%, FP 4: 6%. The distribution of the FP-classes shows, that 93% of the examined study participants could be assigned to the classes 2 and 3.

The FP classes estimate the RTS-score very well. If the skin type classes are formed using pigmentation after repeated sun exposure, the correlation rises to 0.65 and the correct classification increases to 75%. The boxplot also reveals a good differentiation of the four skin types (figure 1B). However, only 617 of the 655 cases could be assigned to a FP class. Therefore, the good agreement is likely due to a selection bias. The wide range of phenotypic attributes is not represented by the given FP-classes, for example for those participants who burn easily but also tan very well.

In 663 cases, the skin type classes according to Uter could be formed: The following frequencies were seen: U1: 109 (16%), U2: 126 (19%), U3: 285 (43%), U4: 115 (17%), U5 28 (4%). These skin type classes are not very good estimates of the RTS-score. Neither correlation nor classification shows a good agreement with the RTS-score (figure 1C). The single indicators hair color and freckles show a better association to the RTS-score compared to the combined indicators (= U-classes).

As expected, the RTS-score and the SSSE-score show a strong correlation, since the five indicators of the SSSE-score with the categories and loadings strongly approximate to the five indicators of the RTS-score. Thus, a strong correlation of r_s _= -0.88 is calculated. However, after classification of the scores into four classes an agreement of only 61% could be reached. Categorization into three risk classes resulted in an association of rs = -0,71 and a correct classification in 72%. Although correlation is high between the two methods, considerable differences are demonstrated, if risk classes are formed.

## Discussion

In the present study, we examined a new questionnaire for sun sensitivity in an occupational skin cancer screening program. The questionnaire showed good internal consistency and good agreement with the Fitzpatrick classification. This suggests, the new questionnaire is a valid and reliable instrument to measure self-reported sun sensitivity.

An unexpected finding is, that we were unable to find a clear definition of sun sensitivity. Currently, there exists a wide variety of terms, tools, and evaluation methods to describe and to model sun sensitivity in epidemiological studies and in public health programs.

Since sun sensitivity is a construct that cannot be directly observed or measured, there is a need for a simple, valid and reliable tool to measure this important risk factor for skin cancer. To our knowledge such an instrument does not exist. Our study is an attempt to address this need by utilizing a questionnaire that at least offers the standard of being developed on the basis of a consensus by dermatological experts.

The RTS-questionnaire consists of all known indicators for sun sensitivity described in the literature so far. In a previous comprehensive review, it was demonstrated that along with the tendency to sunburn and the ability to tan, hair color, skin color and eye color as well as the development of freckles are critical for the assessment of sun sensitivity [4]. The RTS-questionnaire comprises four additional items. The tendency for sunburn is characterized by three further items (reaction to sun exposure of the face, time until sunburn, extent of sunburn). The ability to tan is subdivided into pigmentation after a single sunbath and pigmentation after repeated sunbaths. Further questions to consider are whether age and gender do interact with sun sensitivity. There is an ongoing discussion about sun sensitivity of young people under the age of 20 [2,28]. However, basic data for sun sensitivity for different age groups are rare or missing [11]. Recently, in a case-control study a constitutional susceptibility score was created based on regression coefficients of a logistic regression model that included age [3]. Gender differences for sun sensitivity were found in other studies due to lighter skin color in females [20,29]. In our study, the proportion of females rating their untanned skin as pale was 61% compared to 51% in male. Although the 95% confidence intervals for the means of the RTS-score for females and males did not overlap in our study, the magnitude of the difference is small and therefore clinically not relevant. The difference may reflect an actual gender difference of sun sensitivity, but may also be due to a difference in self-perception between males and females. Young women especially may consider their skin tone more often as too light since a tanned skin has a strong image of increased attractiveness [30]. Further studies on larger populations are necessary to analyze the interaction of sun sensitivity with gender and age.

There are limitations that apply to the interpretation of our results. Variation in sun sensitivity differs from person to person on a continuous scale. The currently available indicators for sun sensitivity are on an ordered ordinal scale. Walters et al. described the preconditions that have to be fulfilled to justify that limited discrete values can be treated as continuous [27]. Since the underlying scale in our study is continuous, the discrete RTS scale has 31 categories and dermatological experts have constructed the ordered ordinal answers of the RTS questionnaire, we are confident that the RTS-questionnaire meets the preconditions as described by Walters et al. [27]. Each study participant who completed the RTS-questionnaire could be assigned an RTS-score. In contrast to the RTS-score not every participant could be assigned a FP-class. Thus, the use of the RTS-score avoided a selection bias. Given the good agreement found in our study with the Fitzpatrick classes, this model of sun sensitivity is an improvement on the current standard. However, the RTS-score is still an imperfect measure and further development of the measurement instruments is necessary.

The assessment of sun sensitivity by questionnaires in contrast to interviews is a frequent topic of discussion in studies. It was demonstrated, that age, sex and former sunburn episodes can influence the answers in questionnaires [11,31]. Furthermore a tendency to underestimate sun sensitivity has been described [11,32]. The examined participants in our study are not a random sample from the general population. Employees of several companies were examined and employees are usually healthier than the general population (healthy worker effect). Since people with a higher risk for skin cancer attend skin cancer screenings more frequently, the examined population is not representative for the working population either. The study participants filled in the questionnaire directly before having a skin cancer screening performed, which may lead to a systematic error.

The comparison of the RTS-score with various methods has several limitations. From the present data, sun sensitivity risk groups (e.g. classes) as defined by other investigators could only be formed approximately. Thus, the other methods may actually represent sun sensitivity differently than delineated in our questionnaire. In consideration of these limitations, however, the preconditions for a comparison are given.

The problem with the Fitzpatrick classes is related to the degree of objectivity in the assignments. The phenotypic variety even in the white population is so large, that many people cannot be assigned to one of the four classes or the assignment to a certain class is based on a subjective decision. In our study 6% of the cases could not be assigned to a Fitzpatrick class. In other studies up to 40% of the cases could not be assigned to a Fitzpatrick class [8]. Furthermore, the measurement is not standardized, so that the skin type assessment according to Fitzpatrick subsumes a large number of different measurements [4,8,10,12,33]. The FP-classes in epidemiological studies or in prevention programs lead either to selection bias or to arbitrary assignment of risks and should therefore no longer be used.

The Uter-score is based on a phenotypic model, which only takes the attributes hair color and freckles into account. In a large study population of 3765 children five skin type classes are formed by a CHAID-analysis after standardized interviews [19]. The model is not only based on a consensus, but on calculated data of a model. The classes show a clear differentiation of the risk groups in a logistic regression, if the target variable is dichotomized (sunburn = FP skin type I + II, pigmentation = FP skin type III and IV). The skin type classes of Uter can only insufficiently represent sun sensitivity in comparison to the RTS-score. An RTS-score of 22 can, for example, be assigned to every Uter skin type class (figure 1C). The differences between the RTS-score and the Uter classes are very large. There are several possible explanations for these differences: The Uter classification is based on a statistical procedure, including the FP-classes in a dichotomized form. Therefore, this model may include the known problems of the Fitzpatrick classification. Further reasons are possibly the different ways of data collection. Specifications of the study population and/or the actual risk classes are represented better by the Uter classes – meaning that in comparison to the traditional indicators the Uter classification results in a different and maybe superior risk profile. It is interesting to note, that the untanned skin color was not considered in the CHAID-analysis. Many authors find that the untanned skin color is an important indicator for sun sensitivity. Assessment of the skin color is frequently used as a reference method [20]. However, we have not come across any study in which the Uter classification is used for the assessment of sun sensitivity.

Guinot developed a score which for the first time quantitatively describes sun sensitivity of the skin by its reaction to UV-rays and by the phenotypic attributes [20]. In that study, the data of 4912 study participants were collected by standardized interviews. In the SSSE-score three phenotypic attributes were included (hair color, skin color, freckles) along with the tendency to sunburn and the ability to tan. By multiple correspondence analysis, the components of the score are described as a linear function. The weights of the components are equal to the "loadings" with the coefficients. The RTS-score in our study correlates very well with the SSSE-score of Guinot (table 4, figure 1D). However, risk classification is different between RTS- and SSSE-score in about 30 to 40% depending on the number of risk classes. Further studies must show whether the weights of the questions need to be changed or whether the additional information actually describes sun sensitivity better.

Our study shows, that the use of only one single indicator (e.g. hair color) for sun sensitivity can lead to misclassification of the risk group in up to 55%. Therefore, it makes sense to use several indicators and summarize the indicators within an index or a score. The inclusion of several indicators comes along with a new problem. In epidemiologic studies on causal factors for skin cancer it is important to correctly model risk factors for skin cancer. If a model is chosen, where phenotypic independent variables, such as freckles, are considered as an independent risk factor for skin cancer, the sun sensitivity questionnaire presented cannot be used since it already contains freckles as an indicator of sun sensitivity.

As demonstrated in our study the selection of indicators differs from investigator to investigator (table 1). However, the selection seems to be arbitrary. The comparison of different scores and models of sun sensitivity resulted in considerable differences in the categorization of risk groups. Therefore, our study supports Gallagher's opinion about "crude" risk stratification for sun sensitivity [21].

In the future, the measurement of the molecular basis of sun sensitivity may replace the current imperfect phenotypic model [34-36]. Until then the current indicators of sun sensitivity must be improved. Investigators should further assess sun sensitivity in large populations, agree on a consensus about the indicators for the measurement of sun sensitivity and standardize the measurement and evaluation. A valid and reliable score for sun sensitivity is an important basis for the scientific analysis of the risk factors for malignant melanoma and other skin cancers.

The introduction of an improved instrument for the assessment of sun sensitivity could also support the prevention of skin cancer. Young people especially could benefit from an individual assessment of their sun sensitivity [5]. Knowledge of one's individual sun sensitivity compared to a reference population could lead to a more risk adapted behavior.

## Conclusion

The RTS-questionnaire is a simple, reliable and valid instrument for the assessment of sun sensitivity. International standardization of sun sensitivity assessment is necessary to strengthen the evidence of epidemiological studies on causal factors of melanoma and other skin cancers.

## Abbreviations

CHAID: Chi-squared Automatic Interaction Detection; FP-classes: Fitzpatrick classes; MCD: Medical Corporate Department; U-classes: Uter classes; RTS: Runder Tisch Solarien (Round Table Sunbeds); SSSE: Sun Sensitivity to Sun Exposure

## Competing interests

This study was not directly funded by any of the following companies that support the occupational skin cancer screening program (BKK Essanelle, Henkel, Cognis, Oleochemicals, Ecolab).

JT received reimbursement for systematic examination of the skin within the skin cancer screening program from the BKK Essanelle. AR is employed by Henkel as head of the Medical Corporate Department. He holds a small number of Henkel shares.

## Authors' contributions

JT und AR designed the study. AR recruited the study participants. JT performed the statistical analyses and drafted the manuscript. JD and BS contributed to the design and manuscript. All authors read and approved the final manuscript.

## Pre-publication history

The pre-publication history for this paper can be accessed here:



## Supplementary Material

Additional file 1 **Appendix: RTS-Questionnaire.** RTS-questionnaire from the "UV-Fibel" published by the German Federal Office for Radiation Protection* [22]. Click here for file
