# Supplementary material for: Assessment of a new questionnaire for self-reported sun sensitivity in an occupational skin cancer screening program
Source: BMC Dermatol. 2008 Oct 24;8:4. doi: 10.1186/1471-5945-8-4 (PMC2582222; doi:10.1186/1471-5945-8-4)
Supplement: Additional file 1 — Appendix: RTS-Questionnaire. RTS-questionnaire from the "UV-Fibel" published by the German Federal Office for Radiation Protection* [22]. [file 1471-5945-8-4-S1.doc]

Appendix: RTS-Questionnaire

RTS-questionnaire from the “UV-Fibel” published by the German Federal Office for Radiation Protection* [22].

|  |  | | |
| --- | --- | --- | --- |
| Questions | | Answers | RTS  Points |
| 1. What is the skin color of your untanned skin? | | Reddish | 1 |
|  |  | Pale | 2 |
|  |  | Light brown | 3 |
|  |  | Brown | 4 |
| 2. Do you have freckles? | | Yes, many | 1 |
|  |  | Yes, some | 2 |
|  |  | Yes, but only a few | 3 |
|  |  | None | 4 |
| 3. How does the skin of your face react to sun exposure? | | Very sensitive, mostly irritation | 1 |
|  |  | Sensitive, sometimes irritation | 2 |
|  |  | Normally sensitive, seldom irritation | 3 |
|  |  | Insensitive, no irritation | 4 |
| 4. How long can you sunbathe without getting a sunburn in early summer at midday with a cloudless sky in Germany or in middle Europe (at sea level)? | | Less than 15 minutes | 1 |
|  |  | Between 15 and 25 minutes | 2 |
|  |  | Between 25 und 40 minutes | 3 |
|  |  | Longer than 40 minutes | 4 |
| 5. How does your skin react to long exposure to the sun? | | Always with sunburn | 1 |
|  |  | Mostly with sunburn | 2 |
|  |  | Often with sunburn | 3 |
|  |  | Rarely or never with sunburn | 4 |
| 6. What kind of sunburn do you develop, when you stay in the sun too long? | | Strong redness, partly painful and blistering, peeling of the skin | 1 |
|  |  | Clear redness, followed by peeling | 2 |
|  |  | Redness, followed sometimes by peeling | 3 |
|  |  | Hardly ever redness and peeling | 4 |
| 7. Do you already experience a tanning effect after sunbathing once for an extended period of time? | | Never | 1 |
|  |  | Hardly ever | 2 |
|  |  | Often | 3 |
|  |  | Almost always | 4 |
| 8. How does your skin tan after repeated sunbathing? | | Hardly ever or never | 1 |
|  |  | Minimal tanning | 2 |
|  |  | Progressive, clear tanning | 3 |
|  |  | Quick and deep tanning | 4 |
| 9. How can you best describe your natural hair color? | | Red up to red brown | 1 |
|  |  | Light blond up to blond | 2 |
|  |  | Dark blond up to brown | 3 |
|  |  | Dark brown up to black | 4 |
| 10. How can you best describe your eye color? | | Light blue, light grey or light green | 1 |
|  |  | blue, grey or green | 2 |
|  |  | Light brown or dark grey | 3 |
|  |  | Dark brown | 4 |

*Question 4 was slightly modified in our study. Our question was: how long can you sunbathe without getting a sunburn at midday with a cloudless sky in Germany?
